# Diagnostic Imaging of Cardiovascular Disease in Small Animals

**DOI:** 10.3390/ani10122392

**Published:** 2020-12-15

**Authors:** Alessia Diana, Carlo Guglielmini

**Affiliations:** 1 Department of Veterinary Medical Sciences, Alma Mater Studiorum-University of Bologna, Via Tolara di Sopra 50, 40064 Ozzano Emilia, BO, Italy; 2 Department of Animal Medicine, Production & Health, University of Padova, Viale dell’Università 16, 35020 Legnaro, PD, Italy

## 1. Introduction

Cardiovascular disease (CVD) has always been an important field of application for diagnostic imaging in small animal practices and, vice-versa, diagnostic imaging has greatly expanded the diagnostic capabilities of veterinary clinicians dealing with CVD. 

In addition to physical examination and electrocardiography, cardiac imaging offers unique opportunities to understand and investigate cardiovascular function and dysfunction. Among the different imaging modalities, each with specific advantages and disadvantages, some focus primarily on cardiac structure and anatomy and others are mainly concerned with cardiac physiology and function.

## 2. From Thoracic Radiography to Echocardiography 

For several decades in the last century, thoracic radiography and fluoroscopy, with or without the use of contrast medium (i.e., contrast angiography), were the imaging techniques of choice to investigate dogs and cats affected by congenital or acquired CVD [1,2]. Radiography is the most commonly available imaging technique in the veterinary clinical setting and allows the simultaneous depiction of the cardiac silhouette, pulmonary and other thoracic vessels, as well as pulmonary parenchyma and pleural space [3]. Therefore, it still plays a pivotal role in diagnosing congestive heart failure (CHF) associated with cardiac disease [3]. 

Starting in the 1970s, the progressively increasing number of available ultrasound technologies, from M-mode via two-dimensional sector and then three-dimensional volume scanning techniques, in combination with various Doppler methods (spectral, color-coded and tissue Doppler) and other advanced techniques (e.g., contrast echocardiography, strain and speckle-tracking imaging), has considerably increased understanding of normal and abnormal cardiovascular anatomy and function [1]. For example, prior to echocardiography, large breed dogs with cardiac arrhythmias or cardiac enlargement and CHF were diagnosed with the generic term of idiopathic or congestive cardiomyopathy [2]. With the advent of diagnostic ultrasound and the capability to more precisely quantify heart chamber size, wall thickness and contractility, as well as blood flow through the heart, specific types of myocardial disease of both dogs and cats were identified, such as dilated, hypertrophic or restrictive cardiomyopathy [2]. The widespread use of echocardiography has made it the dominant cardiac imaging modality in daily veterinary clinical practice [1,2]. Furthermore, the use of echocardiography as the reference diagnostic method has enabled the investigation of the diagnostic accuracy of thoracic radiography for the diagnosis of congenital and acquired CVD in small animals [4,5,6,7]. In addition to the valuable aid in the precise diagnosis of different cardiac diseases, echocardiography enables us to collect useful prognostic information for many of them [8,9,10,11,12,13,14,15].

## 3. From Computed Tomography and Magnetic Resonance Imaging to Artificial Intelligence and Beyond 

In the last decade, the increasing accessibility of computed tomography (CT) and magnetic resonance imaging (MRI) units in small animal veterinary practices has offered additional powerful tools for the in-depth exploration of cardiac anatomy and the assessment of cardiac function. The high temporal and spatial resolution achievable with the modern multidetector-row CT scanners, which can acquire a three-dimensional dataset of the heart and thoracic vessels in a few seconds, allows the depiction of very small or difficult to access normal or abnormal cardiovascular structures (e.g., the coronary arteries) [16]. Furthermore, the use of contrast medium for CT pulmonary angiography represents the technique of choice for the diagnostic confirmation of subtle and challenging vascular diseases (e.g., pulmonary thromboembolism) [17].

The heart and great vessels are very well visualized with MRI because the contrast between cardiovascular tissue and blood is more pronounced than in echocardiography and cardiac CT. Therefore, cardiac MRI is the reference imaging technique for the assessment of cardiac morphology and function and is often used for the evaluation of complex cardiovascular anomalies and specific myocardial disease processes in humans. In companion animals, the early application of MRI includes the precise assessment of cardiac morphology, volume and function, as well as the identification of subtle and challenging changes in poorly accessible cardiovascular structures such as the right ventricle, the pericardium and the heart base in animals with arrhythmogenic cardiomyopathy, mesothelioma, heart base masses and vascular abnormalities, respectively [18]. 

Finally, veterinary cardiologists and radiologists have very recently started the early application of deep learning, an artificial intelligence application that uses computer-aided detection methods with the aim of helping clinicians recognize cardiac or left atrial enlargement on canine thoracic radiographs [19,20].

Cardiovascular imaging has witnessed an explosive expansion of available tools providing detailed information of cardiovascular structures and physiology with noticeable improvement in the diagnosis of CVD. The future directions of cardiac imaging include the increased interconnection among the different imaging techniques and their extended use, not only for diagnostic but also for prognostic purposes in animals with CVD. 
